# Food addiction behaviour and family relationship : about 360 cases

**DOI:** 10.1192/j.eurpsy.2023.1084

**Published:** 2023-07-19

**Authors:** K. Chiha, K. Khemakhem, M. Chaabane, D. Ben Touhemi, J. Boudabous, W. Kammoun, I. HadjKacem, H. Ayadi, Y. Moalla

**Affiliations:** Child Psychiatry, Hedi Chaker University Hospital, Sfax, Tunisia

## Abstract

**Introduction:**

For several years, strong theoretical and clinical links have been established between intra-family relationships and eating disorders.

**Objectives:**

To study intra-family relationships in adolescent with food addictive behaviour.

**Methods:**

This was a cross-sectional, descriptive and analytical study conducted among a sample of adolescents randomly collected in 6 schools in the region of Sfax-Tunisia, during the month of February 2022. The food addiction symptomatology was assessed by the “Dimensional Yale Food Addiction Scale version 2.0 For Children” (dYFAS-C 2.0) and the family attitude was studied by the “Brief Family Relationship Scale” (BFRS) which evaluates three dimensions: cohesion, expressiveness and conflict, each having a separate score. Both scales are validated in Arabic.

**Results:**

The study involved 360 high school students, with a mean age of 16.62 +/- 0.822 years. The sex ratio was 1.09.

The total score for food addiction symptomatology in our sample ranged from 0 to 56 with an average of 16.37 +/- 12.380.

Of the three dimensions of the quality of intra-family relationships studied, conflict had the highest mean score: 25.29+/-9.027.

A high food dependence score was significantly related to these three dimensions of the intrafamily relationship: lack of conflict (p=0.044), cohesion (p=0.011) and expressiveness (p=0.005) presence.

**Conclusions:**

The present study shows that the symptomatology of food addiction is influenced by the quality of the intra-family relationship.

Enhanced perception of the family environment and involvement of the family in possible care can help to prevent the onset of eating disorders and to plan an appropriate intervention.

**Disclosure of Interest:**

None Declared

